# The Influence of Non-Dipping Pattern of Blood Pressure in Gestational Hypertension on Early Onset of Hypertension Later in Life—Single Center Experience in Very-High-Risk Southeast and Central European Country

**DOI:** 10.3390/ijms252011324

**Published:** 2024-10-21

**Authors:** Aleksandra Ilić, Anastazija Stojšić-Milosavljević, Tatjana Miljković, Marija Bjelobrk, Snežana Stojšić, Snežana Tadić, Maja Stefanović, Aleksandra Vulin, Andrej Preveden, Nikola Komazec, Milenko Čanković, Milovan Petrović, Djordje Ilić, Lazar Velicki, Mila Kovačević, Dragana Grković, Aleksandra Milovančev

**Affiliations:** 1 Faculty of Medicine, University of Novi Sad, 21000 Novi Sad, Serbia; aleksandra.ilic@mf.uns.ac.rs (A.I.); anastazija.stojsic@mf.uns.ac.rs (A.S.-M.); tatjana.miljkovic@mf.uns.ac.rs (T.M.); marija.bjelobrk@mf.uns.ac.rs (M.B.); snezana.tadic@mf.uns.ac.rs (S.T.); maja.stefanovic@mf.uns.ac.rs (M.S.); aleksandra.vulin@mf.uns.ac.rs (A.V.); andrej.preveden@mf.uns.ac.rs (A.P.); milenko.cankovic@mf.uns.ac.rs (M.Č.); milovan.petrovic@mf.uns.ac.rs (M.P.); djordje.ilic@mf.uns.ac.rs (D.I.); lazar.velicki@mf.uns.ac.rs (L.V.); mila.kovacevic@mf.uns.ac.rs (M.K.); 2 Institute of Cardiovascular Diseases of Vojvodina, 21208 Sremska Kamenica, Serbia; snezana.stojsic@ikvbv.ns.ac.rs (S.S.); nikola.komazec@ikvbv.ns.ac.rs (N.K.); dragana.grkovic@ikvbv.ns.ac.rs (D.G.); 3 Department of Obstetrics and Gynecology, Clinical Center of Vojvodina, 21000 Novi Sad, Serbia

**Keywords:** gestational hypertension, hypertension, outcomes, blood pressure pattern, endothelial dysfunction

## Abstract

Gestational hypertension (GH) and preeclampsia (PE) are associated with the onset of hypertension. This study aimed to investigate whether the blood pressure (BP) pattern in GH is associated with the prevalence of hypertension later in life. In this prospective cohort study pregnant women screened for GH underwent medical history, laboratory analysis, ambulatory blood pressure monitoring (AMBP), and transthoracic echocardiography (with left ventricular global longitudinal strain (LVGLS)) assessment. Overall, 138 GH (67 non-dippers and 71 dippers), 55 preeclamptic, and 72 normotensive pregnant controls were included. Women were followed in the postpartum period, first after 6 weeks and later on, for the occurrence of hypertension. The median follow-up was 8.97 years (8.23; 9.03). Non-dippers and PE compared with normotensives and dippers had a higher prevalence of hypertension onset (*p* < 0.01), as well as significantly reduced absolute values of LVGLS during pregnancy, after delivery, and at the time of onset of hypertension during follow-up (*p* < 0.01). Night-time diastolic BP, LVGLS, age, and left ventricular ejection fraction were the strongest predictors of postpartum onset of hypertension. The non-dipping BP pattern in GH was significantly associated with the onset of hypertension later in life, as well as with decreased systolic function.

## 1. Introduction

The age at which women are having their first child is increasing, leading to a higher incidence of cardiovascular (CV) risk factors and CV diseases, which are the primary cause of maternal mortality in Europe. Around 10–15% of pregnant women experience hypertension. Hypertensive disorders in pregnancy (HDP) are a major cause of maternal and fetal morbidity, accounting for 15–33% of total maternal mortality and being a leading reason for pregnant women to be hospitalized [1]. Additionally, it is known that HDP is a risk factor for the development of hypertension later in life, but also for ischemic heart disease and stroke [1]. Two HDP entities, gestational hypertension (GH) and preeclampsia (PE), are characterized by specific pathophysiological mechanisms. A recently published study highlighted the connection between alterations in angiogenic factors of placental origin and abnormal placentation in early pregnancy [2]. Also, increased angiogenic factors and decreased proangiogenic factors lead to maternal hypertension and glomerulopathy [3]. Thus, their mutual ratio, which is increased, may be associated with placental ischemia and intrauterine fetal growth restriction (IUGR) [4,5]. The connection between cardiovascular diseases and angiogenic factors has been proven [6]. These markers play a crucial role in vascular remodeling, which is a compensatory mechanism in many cardiac disorders in the non-pregnant population [7], such as myocardial ischemia and heart failure [7,8]. It is known that, in contrast to normotensive pregnancy, preeclampsia is characterized by increased peripheral vascular resistance and reduced cardiac output, as well as reduced perfusion of the mother’s organs and the placenta, which is the reason for complications in pregnancy, both maternal and fetal. Several years ago, it was emphasized that proteinuria (precisely due to the complexity of the pathophysiological mechanism) is not the only criterion for establishing a diagnosis of preeclampsia. For these reasons, the term non-proteinuric preeclampsia was introduced, which implies the presence of gestational hypertension and signs of reduced organ perfusion, even in the absence of proteinuria [9,10,11]. Endothelial dysfunction is the common denominator of both preeclampsia [12] and ischemic heart disease [13], as well as non-dipping blood pressure pattern [14,15,16,17].

This study aimed to investigate whether the blood pressure pattern in gestational hypertension is associated with the prevalence of hypertension later in life. To the best of our knowledge, this is the first study on the impact of the non-dipping pattern of blood pressure during gestational hypertension on the onset of hypertension later in life.

## 2. Results

The median age for all participants was 31 (26; 35) years, and non-dipper and PE groups were significantly older compared with the controls. Basic patient characteristics are presented in Table 1. The median gestational age was 34 (31.5; 36) weeks. Controls had significantly lower body mass index (BMI) compared with all groups, *p* < 0.01. There were no significant differences in median systolic blood pressure (SBP) during the day between the hypertensive groups; however, there was a significant statistical difference in median nocturnal BP—both systolic and diastolic, mean arterial pressure (MAP), and daily diastolic blood pressure (DBP). Heart rate did not differ between groups.

In Table 2, echocardiographic parameters are presented. Left ventricular filling pressure E/e’ was statistically significantly higher in GH and PE compared with controls (*p* < 0.01), while there was no significant differences between hypertensive groups. Dippers had a significantly larger left ventricular end-diastolic volume compared with controls (*p* < 0.01) and compared with non-dippers and PE (*p* < 0.01). The non-dippers and PE group had lower ejection fraction (EF) and cardiac output (CO) index. The left ventricular mass index was significantly higher in hypertensive pregnant women compared with controls (*p* < 0.01). The absolute values of left ventricular global longitudinal strain (LV GLS) were lower in the group of PE and non-dippers, with statistical significance compared with normotensives and dippers (*p* < 0.01). Six months after delivery, LV GLS was significantly lower in PE compared with non-dippers (*p* = 0.03), as well as at follow up in the moment of hypertension onset (*p* = 0.02).

**Figure 1:**
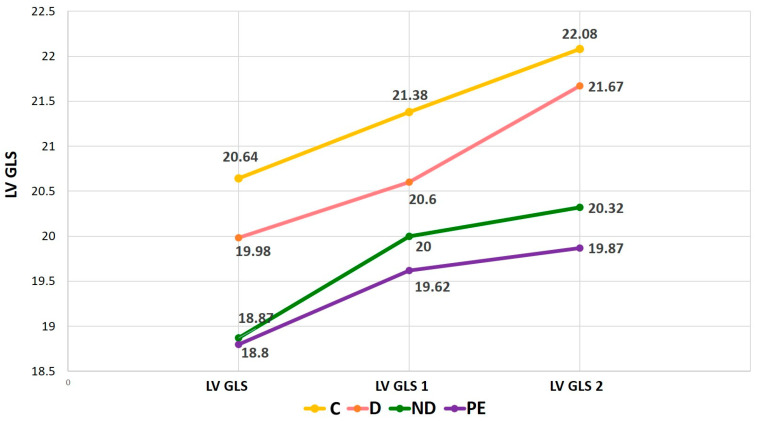
Left ventricular global longitudinal strain values over time. Legend: LV GLS—global longitudinal strain of the left ventricle during pregnancy; LV GLS 1—global longitudinal strain of the left ventricle 6 weeks postpartum; LV GLS 2—global longitudinal strain of the left ventricle in postpartum follow-up; C—controls; D—dippers; ND—non-dippers; PE—preeclampsia.

Figure 1 shows the LV GLS values over time in the full cohort. The LV GLS values at follow-up (GLS 2) for those who became hypertensive were recorded at the time of diagnosis of hypertension, and for the others, at the last control at follow-up. It is very interesting that, at follow-up after delivery, the absolute values of LV GLS were, although in reference values, significantly reduced in women who were non-dippers during GH, compared with both normotensive and dippers (*p* < 0.01), but higher when compared with PE (*p* = 0.02).

The Receiver operating characteristic (ROC) curve showed that LV GLS can be a good marker for the prediction of later onset of hypertension (HTA) The corresponding area under the ROC curve was 0.952 (0.924–0.979) and the best obtained threshold was −18.7 (87.2% sensitivity, 89.5% specificity, *p* < 0.01).

### 2.1. Outcomes of Pregnancy

Preterm delivery and IUGR were significantly more prevalent in non-dipper and PE groups compared with controls (*p* < 0.01). Also, non-dippers and PE had significantly lower Apgar scores and birth weight compared with control and dippers (*p* < 0.01), but there was no significant difference between non-dippers and PE The highest prevalence of Caesarean section was in non-dippers followed by PE, dippers, and controls, with a significant statistical difference between the groups (*p* < 0.01) (Table 3).

### 2.2. Primary Outcome-Hypertension

**Figure 2:**
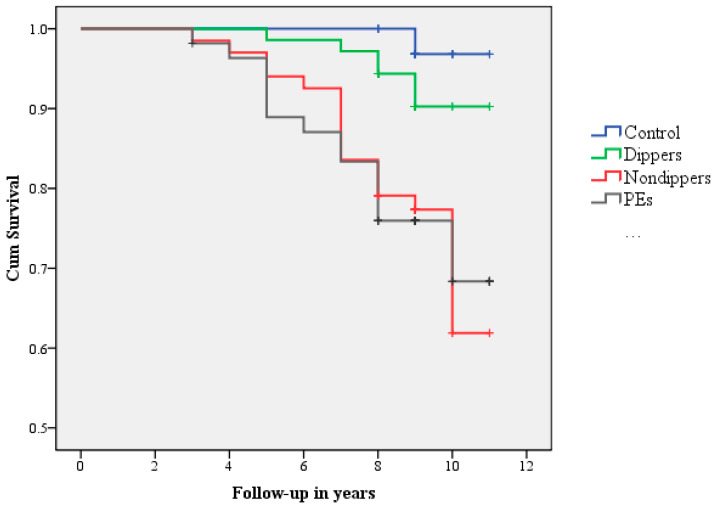
Cox regression of onset of hypertension.

After a median follow-up of 8.97 years (8.23; 9.03), hypertension was diagnosed in 38 (14.3%) participants: 2 (2.8%) in the control group vs. 6 (8.5%) in the dipper group, and 16 (23.9%) in the non-dipper group vs. 14 (25.5%) in the PE group; *p* < 0.01. The average time to event (onset of postpartum hypertension) was 7 (4.97–8.01) years. Although the shortest time to the event was observed in non-dippers 6.99 (5.5–7.97) and PE 6.44 (4.91–7.90), there was no statistically significant difference between the groups, except for dippers 8.13 (7.03–8.99) vs. 6.44 (4.91; 7.90) preeclampsia (*p* = 0.03). (Figure 2). The median age of the women at the time of hypertension onset was 38.06 (38.01; 38.12) in controls, 39.51 (37.99; 41.03) in dippers, 40.49 (35.99; 41.51) in non-dippers, and 35.99 (35.99; 41.91) in PE. There were no significant differences between groups.

Based on follow-up and the development of hypertension in a later period, the parameters at screening were compared between women who experienced hypertension and those who remained normotensive (Table 4).

Women that developed hypertension had significantly reduced absolute values of LV GLS during pregnancy: −17.9 (−17.5; −18.1) compared with normotensive −19.8 (−19.0; 20.8), (*p* < 0.01), but also after delivery: −18.9 (−18.5; 19.1) vs. −21.0 (−20.6; 21.7) (*p* < 0.01) and at the time of onset of hypertension during follow up −19.55 (−18.5; 20.8) vs. −21.4 (−20.2; 22.0) (*p* < 0.01). They were also older, had a higher BMI, and had higher values of both systolic and diastolic blood pressure both during the day and at night. Also, echocardiographic parameters showed worse left ventricular systolic and diastolic functions, as well as a significantly higher left ventricle (LV) index (Table 4).

Women who developed hypertension in the postpartum follow-up period compared with those who remained normotensive were significantly more likely to give birth prematurely (*p* < 0.01) and have a child with intrauterine growth restriction (*p* < 0.01), without a significant difference in the method of delivery (*p* = 0.86). The independent predictors of primary outcome onset of hypertension revealed by univariate Cox regression were age, average daily and nocturnal BP, both systolic and diastolic, MAP, LV mass index, preterm delivery, IUGR, Apgar score, LV GLS, and LV EF (Table 5). Multivariate Cox regression showed that in the model with age, diastolic night-time BP, LV GLS, and EF were the strongest independent predictors of hypertension.

## 3. Discussion

Our research revealed that a non-dipping pattern of blood pressure in gestational hypertension is significantly associated with the onset of hypertension later in life. Also, we showed that increased values of LV mass index, preterm delivery, IUGR, decreased values of Apgar score, and deterioration of LV systolic function are predictors of hypertension occurrence in the future. Additionally, all mentioned parameters were significantly more prevalent in non-dippers and in PE compared with dippers and normotensive pregnant women. In addition, the time of onset of hypertension was shortest in the non-dipper and preeclampsia group. Several studies have shown an association between hypertension in pregnancy and cardiovascular diseases later in life. Although the exact underlying mechanism is not entirely clear, it is assumed that angiogenic peptides could be a shared pathway. It has been proven that elevated values of these peptides lead to endothelial dysfunction, which results in placental ischemia, hypertension, and multiorgan damage, as well as being associated with atherosclerosis [18,19]. As we stated in the Introduction, increased values of circulating soluble fms-like tyrosine kinase 1 (sFlt-1) lead to a decrease in values of proangiogenic factors, such as placental growth factor (PIGF) and vascular endothelial growth factor (VEGF), and an increase in their mutual ratio sFlt-1/PlGF may be responsible for this connection [2,3,4,5,6,7,8]. The findings of our research, which show that the non-dipping pattern of BP in GH is associated with higher prevalence of hypertension later in life, could be comparable to the fact that the non-dipping pattern of BP in the general (non-pregnant) population is a more significant predictor of adverse cardiovascular events than the blood pressure level itself [20,21,22,23,24]. This can be explained by a common underlying pathophysiological mechanism that is a consequence of endothelial dysfunction, common to the non-dipping pattern of BP, IUGR, preeclampsia, and cardiovascular diseases, but also to diabetes and autoimmune and kidney diseases [11,12,13,14,15,16,22,23]. We also showed that increased diastolic nocturnal blood pressure in hypertensive pregnancies is one of the strongest predictors of hypertension later in life. Univariate regression analysis revealed that an increase in night-time BP of 1 mmHg during pregnancy increases the possibility of hypertension by 8.6% in the later period, while the chance of preterm delivery increases by 28.2%. A recent study highlighted that higher values of diastolic blood pressure during pregnancy, but also preterm delivery and low weight of newborns, were associated with higher cardiovascular mortality in those women in the future [18]. The birthweight in our study was the lowest in the non-dipping group (statistically significantly lower compared to dippers and normotensive pregnant women), even lower compared with pregnant women with preeclampsia, although without a statistically significant difference. The results were similar for the Apgar score, both in the first and fifth minutes, which, like preterm delivery and IUGR, were shown in our research to be predictors of the onset of hypertension during follow-up. Both fetal growth restriction and preterm delivery were more prevalent in non-dippers and preeclampsia (with no significant difference between these two groups). Vazquez-Agra et al. showed that an insufficient nocturnal drop in diastolic blood pressure increases the high blood pressure load, which is a main factor in endothelial dysfunction, atherosclerosis, and arterial stiffness [25]. Our research also revealed that women who became hypertensive in the postpartum period had significantly reduced absolute values of LV GLS (in the following text, each comment refers to the absolute value of LV GLS) during pregnancy compared with those who remained normotensive during the postpartum follow-up period. Besides that, it is important to mention that LV GLS values during pregnancy were significantly lower in pregnant women with a non-dipping pattern of blood pressure and those with preeclampsia (no significant difference between these two groups) compared with those with a dipping pattern and normotensive pregnant women. This indicates that left ventricular systolic function is significantly more impaired in non-dippers compared with dippers, which is consistent with what we have previously shown [26]. During the postpartum follow-up, significantly lower values of LV GLS, although in reference values, were still maintained in women who belonged to the non-dipping group compared with the dipping and control groups. However, in that period, a significant difference appeared between the non-dipping group and the group of pregnant women who had preeclampsia, and the lowest values of LV GLS in the postpartum follow-up were observed for the PE group. Recently published research has shown that the non-dipping pattern of BP is also linked to impaired LV mechanics and that LV global longitudinal and circumferential strain, as well as endocardial and epicardial strain, are significantly reduced among non-dippers and reverse dippers [20]. We showed that a decreased value of LV GLS during pregnancy is the strongest predictor for the onset of hypertension later in life, since the multivariate model revealed that a decrease in LV GLS by 1 increases the risk of hypertension by 76%. The cut-off LV GLS value in our study was −18.70. While we are not aware of any research conducted in pregnant women on this topic, it is known that the LV GLS is a significantly better predictor of future adverse cardiovascular events compared with the ejection fraction of the left ventricle [27,28,29,30]. Additionally, a connection between endothelial dysfunction and poor values of LV GLS is known [31]. This could be especially applicable in pregnant women, since it is well known that, due to geometric confounders, strain better reflects systolic function compared with EF [32]. It is known that GH and PE have been associated with the onset of hypertension later in life and that hypertensive disorders during pregnancy double the risk of ischemic heart disease and stroke [1]. Thus, hypertensive pregnant women are at risk of developing cardiovascular disorders in the future. On the other hand, atherosclerotic (CV) diseases are the major cause of morbidity and mortality in Europe, especially in very-high-risk countries [33], while high BP is the most important modifiable risk factor for all-cause and CV disease morbidity and mortality globally [34]. One of the main reasons for such devastating data is the inadequate diagnosis and treatment of hypertension. Pregnant women with hypertension face significant risk as their pregnancies are more likely to be complicated by preeclampsia, eclampsia, and peripartum cardiomyopathy. They also have a higher likelihood of experiencing IUGR and are four times more likely to develop hypertension later in life compared with women who did not have hypertension during pregnancy. Also, hypertensive pregnant women are at increased risk of developing hypertension and preeclampsia in subsequent pregnancies [1]. Recent studies have shown that treatment of hypertension reduces BP values during the night and that conversion of the BP pattern from non-dipping to dipping reduces cardiovascular risk [20,21]. Also, there are assumptions that taking therapy in the evening reduces BP values during the night [35]. Therefore, it should be kept in mind that pregnant women at higher risk, such as those with GH and a non-dipping pattern of BP, are more often controlled and that therapy is prescribed to them adequately, respecting a personalized approach in the treatment of hypertension, which is particularly emphasized in the latest guidelines [36,37]. To the best of our knowledge, this is the first study to examine the impact of a non-dipping blood pressure pattern during gestational hypertension on the occurrence of hypertension later in life, but also explores association between the absence of nocturnal blood pressure drop and lower global longitudinal strain values of the left ventricle, both during pregnancy and in the postpartum period. With all this in mind, it is of crucial importance to perform 24 h ambulatory blood pressure monitoring during pregnancy and determine the blood pressure pattern so that pregnant women who have GH and a non-dipping blood pressure pattern can be checked and controlled more often during pregnancy. All pregnant women who had HDP should be advised on lifestyle changes and regular blood pressure checks after delivery, regardless of whether it has normalized. Particular attention should be paid to women with preeclampsia, as well as those with a non-dipping pattern of blood pressure in gestational hypertension, as they tend to develop hypertension earlier.

Strength of our research. As far as we know, this is the first study on the influence of blood pressure pattern and the function and geometry of the left ventricle in pregnant women with gestational hypertension on the development of hypertension later in life. The advantage of this research is that it included an extremely homogeneous group of pregnant women with gestational hypertension, without any comorbidities and laboratory abnormalities.

Limits of our research. This study is a single-center experience, but from a country with very high cardiovascular risk [33]. Certainly, future research is needed, both in terms of increasing the number of pregnant women and the centers that would be included in the research. In addition, it would be very good and useful to monitor angiogenic biomarkers in pregnant women with a non-dipping blood pressure pattern and compare them with those of pregnant women with a dipping blood pressure pattern in gestational hypertension, as well as those with preeclampsia.

## 4. Materials and Methods

### 4.1. Patient Selection

**Figure 3:**
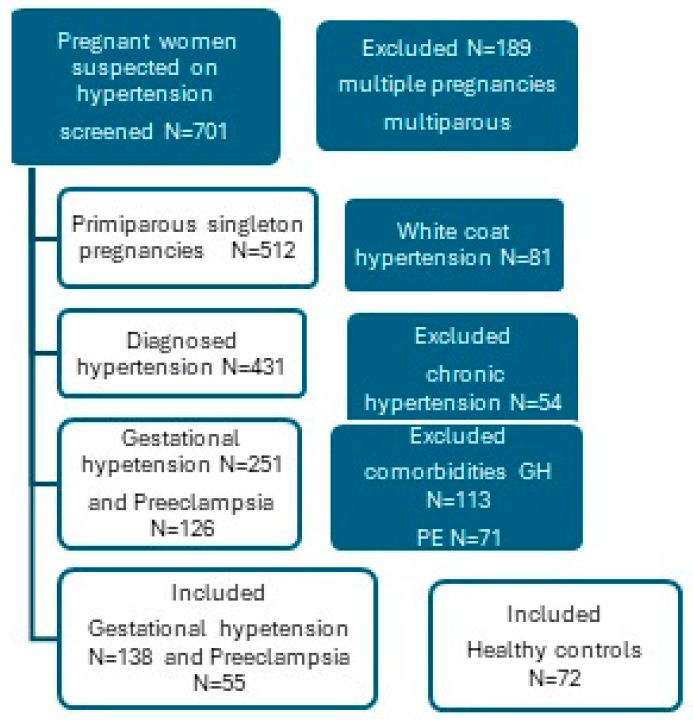
Study flowchart. Abbreviations: PE-preeclampsia.

This prospective cohort study aimed to assess the characteristics and outcomes of pregnant women with gestational hypertension in relation to their blood pressure patterns. Pregnant women referred to cardiology examination for suspected hypertension were screened for eligibility from 1 September 2013 to 31 December 2015. The study flowchart is shown in Figure 3. Detailed examination, medical history, laboratory findings, 24 h ambulatory blood pressure monitoring (AMBP), and transthoracic echocardiography were performed for all patients. Median gestational age was the period in a gestational week when the diagnosis of hypertension was confirmed or rejected, and when ambulatory blood pressure monitoring (ABPM) and transthoracic echocardiogram were performed. GH was defined as BP ≥ 140/90 mmHg that developed after the 20th gestational week (GW) without proteinuria and resolved within 6 weeks postpartum. Preeclampsia (PE) was defined as gestational hypertension (GH) with proteinuria, chronic hypertension was defined as hypertension diagnosed before pregnancy or before the 20th GW [38]. In total 701 pregnant women were screened, of which 512 were primiparous, singleton pregnancies, of which 81 were excluded due to white coat hypertension. Hypertension was confirmed in 431, 54 of them had chronic hypertension, 251 GH, and 126 PE. We excluded women with all known comorbidities (diabetes mellitus, thyroid gland disorders, autoimmune diseases, elevated transaminases, hematological disorders, previously known chronic kidney disease, dyslipidemia, cardiomyopathies, etc.) to obtain a homogenous group. Of the 138 women with gestational hypertension after performing AMBP, 71 met the criteria for a dipping pattern of BP—dippers, and 67 were classified as non-dippers, while 55 women had PE without comorbidities and laboratory abnormalities except proteinuria. None of the women received medications at the time of assessment. If GH was diagnosed, methyldopa was prescribed. Blood pressure values were regularly monitored through home and office blood pressure checks. Additionally, ABPM was performed during pregnancy if needed. Therapy was adjusted if blood pressure values remained high, and nifedipine was added if required. Controls were 72 normotensive primiparous healthy women without comorbidities who voluntarily participated in this research. A total of 265 pregnant women were included in this research.

### 4.2. Study Protocol

#### 4.2.1. ABPM

Ambulatory blood pressure was assessed (ABPM) on the non-dominant arm over a 24 h period using the Meditech Cardio Tens device (Meditech Ltd., Budapest, Hungary). Readings were taken every 20 min from 6 a.m. to 10 p.m., and every 30 min from 10 p.m. to 6 a.m. the next day. Subsequent calculations were performed based on the ABPM readings: average daytime and nighttime systolic blood pressure (SBP) and diastolic blood pressure (DBP). Mean arterial pressure (MAP) was calculated as (SBP + (2 × DBP))/3 [38]. According to the values of day and nighttime BP, a drop of more than 10% in nocturnal arterial blood pressure is referred to as dippers, and those with a smaller decrease are referred to as non-dippers [38]. ABPM was performed on screening to establish/exclude the diagnosis of hypertension, as well as 6 weeks after delivery and during follow-up.

#### 4.2.2. Echocardiography

All participants underwent transthoracic echocardiography in the left lateral decubitus on screening, six weeks after delivery, and during follow-up. Scans were acquired from standard parasternal and apical views using a GE Vivid 9 machine equipped with an M 5S-D, 1.5–4.6 MHz transducer, with simultaneous electrocardiogram monitoring. Three cardiac cycles of uncompressed data were deposited in cine-loop format and analyzed by the investigator, blinded to the characteristics of pregnant women. Chamber quantification, LV myocardial mass (LVmass), and systolic function, as well as LV global longitudinal strain (GLS), were assessed in the parasternal long-axis, short-axis, and apical chamber views according to the recommendations of the American Society of Echocardiography (ASE) [39,40]. Stroke volume (SV) was calculated as the product of the aortic Doppler flow velocity time integral and the cross-sectional area of the LV outflow tract. Cardiac output (CO) was calculated as the product of stroke volume and heart rate (HR) derived from ECG monitoring (CO = SV HR) and then normalized for BSA as the CO index. The GLS values at follow-up (GLS 2) for those who became hypertensive were recorded at the time of diagnosis of hypertension, and for the others, at the last control during follow-up. Diastolic function was evaluated based on the recommendations of the European Association of Echocardiography (EAE) and ASE in the apical 4CH view [41,42]. Transmittal inflow was recorded using pulsed wave Doppler recordings at the mitral valve leaflet tips. The peak velocity of early diastolic filling (E), was measured. The early velocities of the septal end lateral mitral annulus (e’s and e’l) were measured using TDI and then their average ratio e’/a’av was calculated to assess the main parameter of diastolic function—the filling pressure of the left ventricle, E/e’av.

### 4.3. Pregnancy Outcomes

An investigator, who was unaware of the results of maternal echocardiography and ABPM, tracked the development of gestation until delivery to assess the pregnancy outcome (intrauterine growth restriction (IUGR), preterm delivery, and birth weight). IUGR was defined as fetal weight below the 10th percentile for its gestational age [43]. Preterm delivery was defined as delivery before the 37th GW [44].

### 4.4. Postpartum Control

All of the patients were submitted to a clinical evaluation 1.5 to 2 months after delivery in order to assess BP. The participants who were found to be not normotensive according to office blood pressure measurement and AMBP were excluded from this study. Patients were followed until August 2024 for the occurrence of the primary outcome of hypertension onset.

### 4.5. Statistical Analysis

Categorical variables are presented as absolute numbers and percentages. The Kolmogorov–Smirnov test was used to test the normal distribution. Continuous variables are presented as the means and standard deviations or median with interquartile ranges (25th and 75th percentile). Differences between groups were tested via Student’s paired *t*-test or Mann–Whitney test, ANOVA, Wilcoxon, and the chi-square test as appropriate. Cox proportional hazard regression was used to determine independent predictors of hypertension onset, and these were expressed as estimated hazard ratios (HRs) with their corresponding 95% confidence intervals (CIs). Variables found to be statistically significant in univariable analysis were used for the multivariable model. A backward Cox multivariable regression was used to build the model, and *p*-values lower than 0.05 were considered statistically significant. The statistical software Statistica (Statistica 13.5, The Ultimate Academic Bundle, StatSoft Europe GmbH, Hamburg, Germany; university license for the University of Novi Sad) was used for all analyses.

## 5. Conclusions

The non-dipping pattern of BP in GH is significantly associated with the onset of hypertension later in life, but also with decreased systolic function of the LV, both during pregnancy and in the postpartum period. The strongest predictors of postpartum onset of hypertension were age, night-time diastolic BP, LV GLS, and LV ejection fraction.

## Figures and Tables

**Table 1:** Patients’ baseline characteristics, blood pressure, and heart rate obtained by 24 h ambulatory blood pressure monitoring.

Parameter	Controls Median (Q1, Q3)	Dippers Median (Q1, Q3)	Non-Dippers Median (Q1, Q3)	PE Median (Q1, Q3)	P1	P2	P3	P4	P5	P6
Age	29 (25; 33)	32 (27; 35)	32 (27; 35)	33 (27; 35)	0.06	0.03	0.02	0.75	0.45	0.59
GW	36 (34; 38)	33 (31; 35)	33 (31; 35)	33 (31; 35)	<0.01	<0.01	<0.01	0.88	0.66	0.59
BMI (kg/m^2^)	27.16 (26.08; 27.72)	30.84 (28.87; 32.39)	29.05 (26.66; 33.44)	30.49 (28.19; 33.41)	<0.01	<0.01	<0.01	0.08	0.72	0.18
ABPM average SBP daytime (mmHg)	119 (115; 122)	143 (139; 146)	145 (140; 150)	143 (135; 147)	<0.01	<0.01	<0.01	0.06	0.69	0.06
ABPM average SBP night-time (mmHg)	105 (98; 107)	125 (119; 130)	140 (135; 147.5)	135 (125; 143)	<0.01	<0.01	<0.01	<0.01	<0.01	<0.01
ABPM average DBP daytime (mmHg)	71 (69; 75)	87 (85; 89.5)	95 (92; 100)	92 (85; 95)	<0.01	<0.01	<0.01	<0.01	<0.01	<0.01
ABPM average DBP night-time (mmHg)	58 (55; 61)	72 (64; 75.5)	92 (86; 95)	85 (74; 92)	<0.01	<0.01	<0.01	<0.01	<0.01	<0.01
ABPM MAP (mmHg)	87.67 (84.33; 89.5)	106.67 (101.83; 108.33)	111.33 (108; 115.33)	108 (102.17; 113.67)	<0.01	<0.01	<0.01	<0.01	0.07	<0.01
ABPM average HR (beat/min)	88 (82.5; 100)	87 (81; 99)	90 (82; 97)	89 (82; 98)	<0.01	0.49	0.93	0.75	0.34	0.38

Legend: BMI—body mass index; GW—gestational week, ABPM—24 h ambulatory blood pressure monitoring; SBP—systolic blood pressure; DBP—diastolic blood pressure; MAP—mean arterial pressure; HR—heart rate; P1 value—Controls vs. Dippers; P2 value—Controls vs. Non-dippers; P3 value—Controls vs. PE; P4 value—Dippers vs. Non-dippers; P5 value—Dippers vs. PE; P6 value—Non-dippers vs. PE, Q1—25th percentile, Q3—75th percentile. Baseline echocardiographic features are shown in Table 2.

**Table 2:** Echocardiographic parameters during pregnancy and left ventricular global longitudinal strain after delivery and at follow-up.

Parameter	Controls Median (Q1, Q3)	Dippers Median (Q1, Q3)	Non-Dippers Median (Q1, Q3)	PE Median (Q1, Q3)	P1	P2	P3	P4	P5	P6
E (m/s)	0.9 (0.8; 1.04)	0.80 (0.7; 0.9)	0.7 (0.7; 0.8)	0.8 (0.7; 0.8)	<0.01	<0.01	<0.01	<0.01	0.04	0.21
e’s (m/s)	0.12 (0.1; 0.13)	0.09 (0.09; 0.1)	0.09 (0.07; 0.09)	0.09 (0.07; 0.09)	<0.01	<0.01	<0.01	<0.01	0.02	0.98
e’l (m/s)	0.15 (0.13; 0.18)	0.11 (0.10; 0.13)	0.1 (0.09; 0.11)	0.1 (0.09; 0.12)	<0.01	<0.01	<0.01	<0.01	0.14	0.16
E/e’av	6.89 (6.21; 7.92)	8.24 (6.96; 9.28)	8.46 (7.50; 8.75)	8.24 (7.37; 8.75)	<0.01	<0.01	<0.01	0.70	0.87	0.45
LV EDV (mL)	96 (85; 109)	107 (98; 116)	90 (77; 106)	91 (78; 104)	<0.01	0.30	0.25	<0.01	<0.01	0.89
LV ESV (mL)	33.5 (27; 40)	38 (34.25; 42)	35 (31; 40)	35 (30; 37.5)	<0.01	0.24	0.57	<0.01	<0.01	0.61
LV EF (%)	64 (63; 65)	64 (62.5; 66)	63 (60; 64)	63 (61; 64.0)	0.79	<0.01	<0.01	<0.01	<0.01	0.54
CO index (L/min/m^2^)	3 (2.71; 3.28)	3.48 (3–05; 3.77)	2.75 (2.49; 3.02)	2.66 (2.36; 2.87)	<0.01	<0.01	<0.01	<0.01	<0.01	0.13
LV mass index (g/m^2^)	74.51 (72.09; 77.61)	88.23 (83.31; 96.63)	93.75 (85.08; 102.36)	91.47 (77.42; 103.63)	<0.01	<0.01	<0.01	0.02	0.40	0.72
LV GLS	−20.6 (−20.2; −21.1)	−19.5 (−19.2; −20.8)	−19 (−18; −19.7)	−18.9 (−18; −19.4)	<0.01	<0.01	<0.01	<0.01	<0.01	0.80
LV GLS 1	−21.70 (−20.80; −22.00)	−20.20 (−20.00; −21.70)	−20.00 (19.55; −20.45)	−19.8 (−18.95; −20.2)	<0.01	<0.01	<0.01	0.002	<0.01	0.03
LV GLS 2	−22.00 (−21.90; −22.60)	−21.80 (−21.10; −22.00)	−20.00 (−19.80; −20.90)	−19.9 (−19.1; −20.6)	<0.01	<0.01	<0.01	< 0.01	<0.01	0.02

Legend: E—transmittal early peak velocity; e’s—early diastolic mitral annulus septal velocity; e’l—early diastolic mitral annulus lateral velocity; E/e’av—left ventricle filling pressure; LV—left ventricle; EDV—end-diastolic volume; ESV—end-systolic volume; EF—ejection fraction; CO—cardiac output; LV GLS—global longitudinal strain of the left ventricle during pregnancy; LV GLS 1—global longitudinal strain of the left ventricle 6 weeks postpartum; LV GLS 2—global longitudinal strain of the left ventricle at postpartum follow-up; P1 value—Controls vs. Dippers; P2 value—Controls vs. Non-dippers; P3 value—Controls vs. PE; P4 value—Dippers vs. Non-dippers; P5 value—Dippers vs. PE; P6 value—Non-dippers vs. PE, Q1—25th percentile, Q3—75th percentile.

**Table 3:** Pregnancy outcomes.

	Controls N (%) Median (Q1, Q3)	Dippers N (%) Median (Q1, Q3)	Non-Dippers N (%) Median (Q1, Q3)	PE N (%) Median (Q1, Q3)	P1	P2	P3	P4	P5	P6
Preterm delivery	9 (12.5)	9 (12.5)	30 (41.7)	24 (33.3)	1.00	<0.01	<0.01	<0.01	<0.01	1.00
IUGR	2 (2.4)	10 (11.9)	41 (48.8)	31 (36.9)	0.02	<0.01	<0.01	<0.01	<0.01	0.71
Cesarean section	22 (15.8)	35 (25.2)	43 (30.9)	39 (28.1)	<0.01	<0.01	<0.01	<0.01	<0.01	<0.01
Apgar score 1st minute	9 (9; 10)	9 (8; 10)	8 (7; 8.5)	8 (6; 9)	<0.01	<0.01	<0.01	<0.01	<0.01	0.59
Apgar score 5th minute	10 (10; 10)	10 (10; 10)	9 (8; 10)	9 (8; 10)	<0.01	<0.01	<0.01	<0.01	<0.01	0.90
Birth weight (g)	3330 (2890; 3660)	2900 (2660; 3350)	2380 (2090; 3150)	2500 (2170; 3125)	<0.01	<0.01	<0.01	<0.01	<0.01	0.52

IUGR—intrauterine growth restriction; P1 value—Controls vs. Dippers; P2 value—Controls vs. Non-dippers; P3 value—Controls vs. PE; P4 value—Dippers vs. Non-dippers; P5 value—Dippers vs. PE; P6 value—Non-dippers vs. PE, Q1—25th percentile, Q3—75th percentile.

**Table 4:** Characteristics of women during pregnancy who developed hypertension compared with normotensive in follow-up.

Parameters During Pregnancy	Onset of Hypertension in Folllow-Up N (%) Median (Q1, Q3)	Normotensive in Folllow-Up N (%) Median (Q1, Q3)	*p*
Age	33 (30; 35)	30 (26; 35)	0.02
BMI (kg/m^2^)	29.38 (27.55; 33.39)	28.96 (27.04; 31.96)	0.04
ABPM SBP daytime (mmHg)	145 (140; 149)	137 (122; 146)	<0.01
ABPM SBP night-time (mmHg)	139 (130; 143)	125 (105.5; 135)	<0.01
ABPM DBP daytime (mmHg)	92 (91; 95)	85 (75; 92)	<0.01
ABPM DBP night-time (mmHg)	72 (60.5; 83)	90 (80; 92)	<0.01
ABPM HR (bp/min)	87 (82; 99)	88 (82; 98)	<0.01
ABPM MAP (mmHg)	109.67 (108; 112.67)	104 (89.67; 110.67)	<0.01
E/e’av	8.57 (7.37; 8.57)	8 (6.67; 8.57)	<0.01
CO index	2.86 (2.51; 3.02)	2.99 (2.66; 3.39)	0.07
LV mass index (g)	97.69 (79.16; 94.40)	84.07 (75.06; 94.40)	<0.01
LV EDV (mL)	94 (85; 108)	96 (85; 109)	0.93
LV ESV (mL)	35 (32; 40)	36 (30; 40)	0.85
LV EF (%)	62.70 (57.89; 65.88)	63.11 (61.76; 64.80)	0.38
LV GLS	−17.9 (17.5; 18.1)	−19.8 (−19; −20.8)	<0.01
LV GLS 1	−18.90 (−18.50; 19.10)	−20.60 (−20.00; −21.70)	<0.01
LV GLS 2	−21.40 (−20.20; −22.00)	−21.40 (−20.20; −22.00)	<0.01

BMI—body mass index; ABPM—24 h ambulatory blood pressure monitoring, SBP—systolic blood pressure; DBP—diastolic blood pressure; MAP—mean arterial pressure; HR—heart rate; E/e’av—left ventricle filling pressure; LV—left ventricle; CO—cardiac output; EDV—end-diastolic volume; ESV –end-systolic volume; EF—ejection fraction; LV GLS—global longitudinal strain of the left ventricle during pregnancy; LV GLS 1—global longitudinal strain of the left ventricle 6 weeks postpartum; LV GLS 2—global longitudinal strain of the left ventricle at the time of onset of hypertension in postpartum follow-up; IUGR—intrauterine growth restriction, Q1—25th percentile, Q3—75th percentile.

**Table 5:** Univariate and multivariable Cox regression of predictors of hypertension onset.

Variables	Univariate		Multivariate	
	HR (95% CI)	*p* Value	HR (95% CI)	*p* Value
Groups	1.896 (1.374–2.617)	<0.01		
Age	1.082 (1.018–1.150)	0.01	1.075 (1.002–1.154)	0.04
ABPM average SBP daytime	1.061 (1.032–1.091)	<0.01		
ABPM average SBP night-time	1.052 (1.030–1.075)	<0.01		
ABPM average DBP daytime	1.086 (1.048–1.126)	<0.01		
ABPM average DBP night-time	1.086 (1.056–1.117)	<0.01	1.036 (1.004–1.069)	0.02
ABPM MAP	1.090 (1.050–1.132)	<0.01		
LV mass index	1.040 (1.019–1.062)	<0.01		
Preterm delivery	0.282 (0.149–0.533)	<0.01		
IUGR	4.896 (2.501–9.585)	<0.01		
Apgar score 1st min	0.805 (0.678–0.955)	0.01		
Apgar score 5th min	0.640 (0.479–0.855)	<0.01		
LV GLS	0.175 (0.113–0.270)	<0.01	0.240 (0.151–0.382)	<0.01
LV EF	0.690 (0.615–0.774)	<0.01	0.848 (0.759–0.948)	<0.01

ABPM—24 h ambulatory blood pressure monitoring, SBP—systolic blood pressure; DBP—diastolic blood pressure; MAP—mean arterial pressure; LV—left ventricle; IUGR—intrauterine growth restriction; GLS—global longitudinal strain during pregnancy; EF—ejection fraction.

## Data Availability

The data presented in this study are available on request from the corresponding author.
